# Compositional and toxicological investigation of pooled venom from farm-raised *Naja atra*

**DOI:** 10.1590/1678-9199-JVATITD-2021-0040

**Published:** 2022-03-14

**Authors:** Gang Xiao, Junqi Liu, Lingfeng Peng, Yang Yang, Zhiliang Sun

**Affiliations:** 1 College of Veterinary Medicine, Hunan Agricultural University, Changsha, Hunan, China.; 2 Hunan Engineering Research Center of Veterinary Drug, Hunan Agricultural University, Changsha, Hunan, China.; 3 Veterinary Drug Laboratory, Hunan Institute of Animal and Veterinary Science, Changsha, Hunan, China.

**Keywords:** Naja atra, Phospholipase A_2_, Three-finger toxins, Neurotoxicity, Myotoxicity

## Abstract

**Background:**

*Naja atra* is a venomous snake species medically relevant in China. In the current study, we evaluated the composition and toxicological profile of venom collected from farm-raised *N. atra*.

**Methods:**

Venom was collected from third-generation captive bred *N. atra* on a snake farm in Hunan Province, China. The venom was analyzed using sodium dodecyl sulfate polyacrylamide gel electrophoresis and nano-liquid chromatography with electrospray ionization tandem mass spectrometry. In addition, hemolytic activity, median lethal dose, serum biochemical and histopathological parameters were accessed.

**Results:**

*N. atra* venom proteome was dominated by phospholipase A_2_ (46.5%) and three-finger toxins (41.4 %), and a set of common low relative abundance proteins, including cysteine-rich secretory proteins (4.7%), NGF-beta (2.4%), snake venom metalloproteinase (1.5%), glutathione peroxidase (0.6%), vespryn (0.3%), and 5ʹ-nucleotidases (0.2%) were also found. Furthermore, the venom exhibited direct hemolytic activity, neurotoxicity, myotoxicity, and high lethal potency in mice, with a subcutaneous median lethal dose of 1.02 mg/kg. Histopathological analysis and serum biochemical tests revealed that venom caused acute hepatic, pulmonary and renal injury in mice.

**Conclusion:**

This study revealed the composition and toxicity of venom collected from farm-raised *N. atra*, thereby providing a reference for the analysis of venom samples collected from captive-born venomous snakes in the future.

## Background

China possesses an extremely rich and diverse snake fauna, consisting of approximately 209 known species of land and sea snakes. Of these, Chinese cobra *(Naja atra)*, ranked as one of the top ten venomous snakes in the country, is considered one of the most medically important snake species [1]. To meet the increasing demand for snakes and their products, as well as to protect wild snake resources, farm breeding or snake farming is conducted to breed snakes of economic and medical relevance, such as *N. atra.* In Hunan Province, China, farm-raised venomous snakes are a stable source of venom used to produce cosmetics and snake venom hemagglutinase injections.

*N. atra* belongs to the Elapidae family, whose members contain venom that induces neurotoxic, hemolytic, proteolytic, hemorrhagic, and necrotizing activities in experimental models [1-3]. Patients bitten by *N. atra* usually develop necrosis, tremors, blurred vision, tachypnea, arrhythmia, and rarely hemorrhage. Victims may also suffer from acute respiratory, circulatory and renal failure [4]. Recently, several novel proteins with important biological activities have been characterized and isolated from this venom. For example, α-elapitoxin-Na1a, a novel short-chain postsynaptic neurotoxin, was isolated from *N. atra* venom and exhibits relatively strong binding activity with the skeletal muscle nicotinic acetylcholine receptor (nAChR) [5]. Moreover, atrase B, a novel metalloproteinase isolated from *N. atra* venom, may be a potential adjuvant therapeutic drug with application in xenotransplantation owing to the dual anti-complement and anti-coagulation activities [6]. 

Studies [7,8] have previously analyzed the protein composition of *N. atra* venom from Zhejiang and Taiwan, China, and have identified three-finger toxins (3FTx) and phospholipase A_2_ (PLA_2_) as the main components of this venom. Jiang et al. [9]. found that the venom transcriptome of *N. atra* from Zhejiang mainly contains 3FTxs, exhibiting cytotoxicity and neurotoxicity (short chain α-NTX). Moreover, Li et al. [10] assessed the venom proteomic profiles from *N. atra* from Yiwu County, Zhejiang Province, China using four different approaches. Furthermore, previous studies have reported geographic and age-related variations in the proteome and enzymatic and toxicological activities of *N. atra* venom [4,8,11]. Gao et al. [11] found that venom yield of *N. atra* was related to the gender, age and geographic location. 

Studies of other snake species have found differences between venom collected from snakes in captivity and in the wild [12]. Intraspecific variation in venom composition is an important cause of differences in venom function and the clinical pathophysiology of envenomation [13-16]. However, the current critical problem is that the knowledge of venom proteins in snakes from completely different regions is incomplete, and the clinical pathophysiology of patients envenomed by *N. atra* from completely different regions remain unknown. Proteomic approach has been used to characterize *Naja* venoms [17]. Therefore, it is necessary to study the venom proteome, toxicological activities and pathophysiology of *N. atra* from vital areas that include key habitats of this snake.

In the present study, we investigated the venom proteome and toxicity of farmed *N. atra* venom in Hunan Province, China. Our results provide important information on the composition and activities of *N. atra* venom from this province.

## Methods

### Snakes

The snakes from which venom samples were collected from third-generation *Naja atra* captive bred on the Hunan Yongzhou Yishe Science and Technology Industrial, Lingling District, Yongzhou City, Hunan Province, China. The snakes were all hatched at the same time, and the feeding environment and other aspects in the later feeding process were the same for all specimens. The average weight of the snakes was 1.5 kg, with an approximate age of one year. A total of 20 snakes (10 females and 10 males) were maintained at constant temperature and humidity conditions (25 ºC, 60%) and fed with thawed rodents.

### Snake venom

Snakes were provided with water for a week before venom collection; during this period, food was not provided to the snakes. Venom extraction was performed during July-August in a glass beaker covered with a thick plastic sheet and immersed in an ice bath. July-August was the peak period for venom collection in the snake farm, and the interval between each collection was 25 days. The freshly collected venom samples was pooled, freeze-dried in a vacuum dryer and stored at -20 ºC.

### Mammals and reagents

Male and female Kunming mice (20 ± 0.7 g; licensed: SCXK [Xiang] 2016-0002) and rabbits (male; approximate weight: 2.0 kg) were purchased from Hunan SJA Laboratory Animal, Hunan, China. Kits (batch number: 2200424) for analysis of serum creatinine (SCR), urea nitrogen (UN), aspartate aminotransferase (AST), alanine aminotransferase (ALT), and creatine kinase (CK) levels were purchased from Jiangsu Sinnowa Medical Science & Technology, Jiangsu, China. Sodium dodecyl sulfate polyacrylamide gel electrophoresis (SDS-PAGE) gels were purchased from Sigma-Aldrich (St. Louis, MO, USA), and protein endonuclease trypsin (sequencing grade) was purchased from Promega (Madison, WI, USA). Other chemicals for proteolysis were purchased from Sigma-Aldrich.

### SDS-PAGE

SDS-PAGE was done as described by Laemmli [18]. The Spectra Multicolor Broad Range Protein Ladder (11-245 kDa) (Beijing Solarbio Science &Technology, Beijing, China) was used for calibration. Crude venom (20 µg) was loaded onto a 15% gel, and electrophoresis was performed under reducing conditions at 75 V for 30 min, followed by 110 V for 2 h. Subsequently, the gels were stained with Coomassie Brilliant Blue R-250 (Beijing Solarbio Science &Technology, Beijing, China) and the protein bands were visualized and documented using a gel imaging system (Bio-Rad, USA).

### Identification of *N. atra* venom proteins

Sample preparation

Venom was dissolved in ultrapure water and the concentration was adjusted to 1 mg/mL. Subsequently, the sample was centrifuged (3,000 × *g;* 10 min, 4 °C), and the supernatant was used for further analysis. Venom samples were subjected to treatments as per protocols described by Shevchenko et al. [19]. The sample solution was first denatured in 8 M urea, the disulfide linkages were reduced with 10 mM dithiothreitol, and all cysteine residues were alkylated using 55 mM iodoacetamide in the dark at 37 ºC for 45 min. The sample was then subjected to cleaning in a C18-based spin column (Thermo Fisher, USA) and was digested using sequencing-grade modified trypsin (Promega, USA) with a digestion buffer (ammonium bicarbonate 100 mM, pH 8.5). The peptides obtained after digestion were dried in a Speed Vac dryer (Thermo Fisher, USA). The dried sample was then resuspended in 2% acetonitrile, 97.5% water, and 0.5% formic acid and analyzed using the nano-LC-ESI-MS/MS system.

Nano-liquid chromatography-electrospray ionization-mass spectrometry (nano-LC-ESI-MS/MS) analysis

Nano-LC-ESI-MS/MS analysis of the digested protein samples was performed using a high-pressure liquid chromatography (HPLC) system (Agilent, USA) with a reverse phase C18 column (inner diameter: 75 µm; length: 8 cm; particle size: 3 μM; and pore size: 300 Å). The injection time was 20 min. HPLC solvent A comprised 97.5% water, 2% acetonitrile, and 0.5% formic acid, and solvent B contained 9.5% water, 90% acetonitrile, and 0.5% formic acid. The time required for the gradient to run from 2% solvent B to 90% solvent B was 60 min, in addition to 20 min for sample loading and 20 min for column washing. The column flow rate was approximately 800 nL/min after the completion of splitting, and the typical injection volume was 3 µL.

The HPLC system was coupled to an LTQ linear ion trap mass spectrometer (Thermo, USA) such that the sample eluted from the HPLC column was directly subjected to ionization by ESI before sample entry into the mass spectrometer. The ionization voltage was optimized before the conduction of every step and maintained within a range of 1.2-1.8 kV. The capillary temperature was set at 110 °C. The mass spectrometer was used in a data-dependent mode to acquire MS/MS data via a low-energy collision-induced dissociation (CID) process. The default collision energy was 33% and the default charge state was 3. One full scan using a microscan with a mass range of 550-1800 Da was acquired; subsequently, one MS/MS scan of the most intense ion was acquired, which included a full mass range and three microscans. The dynamic exclusion feature was set as follows: repeat count of 1 within 0.3 min and exclusion duration of 0.4 min. The exclusion width was 4 Da.

### Database search and validation

Mass spectrometric data were used to search against the UniProt protein database with ProtTech’s ProtQuest software suite (Norristown, PA, USA). The parameters for data acquisition and processing were as follows. Data acquisition parameters: instrument, LTQ; software, Xcalibur 2.0; and centroid mode minimum MS signal for precursor ion: 5 × 10^4^ counts. Data processing parameters: software, ProtQuest 2.0; signal-to-noise-ratio, ≥ 5; De-isotoped, yes; filtered, yes; missed cleavages, ≤ 5; modifications, Cys +57; and database, Serpentes protein database from UniProt (accession date: November 23, 2018). Validation parameters: for proteins identified based on 1 peptide (score ≥ 15); for proteins identified with more than 1 peptide (score ≥ 13).

### Protein quantitation

Protein abundance was calculated using the label-free protein quantitation method described by Griffin et al. [20]. The scoring function was based on the MS abundance recorded in both MS and MS/MS data sets, spectral count (SC; number of MS/MS spectra per peptide), non-duplicate peptide number (PN), and fragment ion (MS/MS) intensities. The normalized spectral index (SI_N_) was calculated for venom solution using the following equation:

$${SI}_{N}=\left[\sum_{k=1}^{pn}{\left(\sum_{j=1}^{sc}i_{j}\right)}_{k}\right]/\left[\sum_{j=1}^{n}{SI}_{j}\right]/L$$

where pn represents the PN, sc represents the SC, i represents the fragment ion intensity of peptide k, j represents the jth spectral count of sc total spectral counts for peptide k, n represents the total number of proteins identified, and L represents the number of amino acids in a protein. 

### Hemolytic activity

The in vitro hemolytic activity of the venom was determined as described by Accary et al. [21], with slight modifications. Blood was collected from healthy rabbits into tubes without anticoagulant, and the erythrocytes were separated by centrifugation (2,000 × g, 5 min). Before analysis, the erythrocytes were washed three times with normal saline and then incubated with increasing normal saline. The erythrocytes were prepared as described above and aliquots were incubated with increasing concentrations (10-100 μg/mL) of venom for 60 min, after which the tubes were centrifuged and the absorbance of the supernatant read at 530 nm. The extent of hemolysis was expressed as a percentage of the absorbance obtained by incubating erythrocytes with distilled water (positive control, 100%). The negative control consisted of erythrocytes incubated with 0.9% saline alone.

### Venom lethality

Mice were injected subcutaneously with venom at doses of 0.5, 0.7, 1.1, 1.7 and 2.5 mg/kg (10 mice/dose) in the dorsal region of the neck in a fixed volume of 100 uL of saline solution. The general condition of the mice, including the manifestations of envenomation and number of deaths, was monitored over the next 24h, during which the animals had free access to food and water. All mice were necropsied to assess the internal venom-induced pathological alterations. The median lethal dose (LD_50_) was calculated using probit analysis [22].

### Histopathological analysis

Kunming mice were subcutaneously injected with an LD_50_ of venom. Randomly selected mice were killed by cervical dislocation at 6, 12, and 24h (n = 3), and their liver, lungs, kidneys, and heart were dissected immediately and fixed using a fixative solution (Wuhan Servicebio Science &Technology, Beijing, China). Tissue sections 5 μm thick [Indicate thickness] were stained with hematoxylin and eosin (H&E) and examined under a microscope.

### Serum biochemical tests

Kunming mice were injected s.c. with one LD_50_ of venom whereas control mice received the same volume of saline. When the venom-injected mice showed signs of envenomation (~1 h after injection), blood samples were collected from the orbital plexus into tubes without anticoagulant and the mice then killed by cervical dislocation. The blood samples were centrifuged (2,800 x g, 4 ℃) and the resulting serum was used for the quantification of ALT, AST, UN, SCr and CK using commercial assay kits.

### Statistical analysis

Experimental data are presented as mean ± SEM. Statistical analyses were performed using the GraphPad Prism 5 software (La Jolla, CA, USA). Two-tailed Student’s t-test and analysis of variance were used to analyze different groups, with P < 0.05 indicating significance.

## Results

### Characterization of N. atra venom proteome

**Figure 1.**
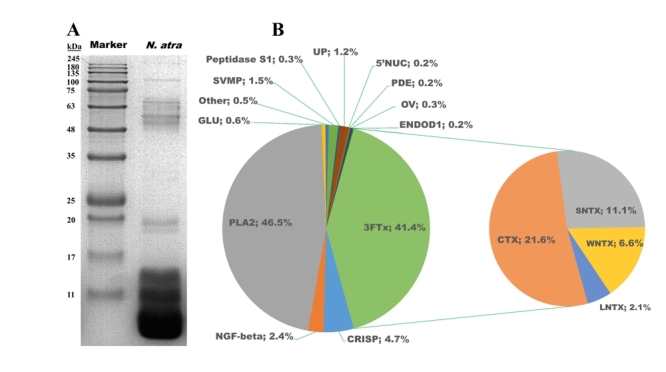
SDS-PAGE and proteomes of N. atra venom. **(A)** Protein separation of N. atra venom (20 μg) on 15% SDS-PAGE under reducing conditions. **(B)** Composition of N. atra venom according to protein families, expressed as percentages of the total protein content. N. atra: Naja atra; PLA _2_: phospholipase A_2_; 3FTx: three-finger toxin; CRISP: cysteine-rich secretory protein; NGF-beta: venom nerve growth factor; SVMP: snake venom metalloproteinase; PDE: phosphodiesterase; 5ʹNUC: 5ʹ-nucleotidases; GLU: glutathione peroxidase; UP: uncharacterized protein; SNTX: short-chain α-neurotoxin; LNTX: long-chain α-neurotoxin; WNTX: weak neurotoxin.

Figure 1A illustrates the protein components in venom based on 15% SDS-PAGE under reducing conditions. An intense low-molecular-weight protein band at < 15 kDa, as well as multiple protein bands with molecular weights ranging from 40 kDa to > 100 kDa, could be observed. To identify the major protein components in N. atra venom samples, we conducted a comprehensive proteomic analysis using nano-LC-ESI-MS/MS. A total of 47 different proteins were identified in the venom, belonging to 21 protein families (Table 1); the main toxin protein families were PLA_2_ (45.6%), 3FTx (41.4%), CRISP (4.7%), NGF-beta family (2.4%), and SVMP (1.5%) (Figure 1B). Additional file 1 shows more detailed data on such proteins. 

**Table 1.** Summary of venom proteins of N. atra assigned by protein families.

Family	Protein name	Protein mass (kDa)	Source organism	Database accession	Length	No. of peptide	No. of non-duplicate peptide	Probability	Score	Relative abundance (%)
PLA_2_	Acidic phospholipase A_2_ natratoxin	14.0	Naja atra	A4FS04	119	188	10	99.0%	2745	46.5
3FTx	Cytotoxin KJC3	7.2	Naja sputatrix	P60311	60	91	8	99.0%	1010	21.0
3FTx	Neurotoxin homolog NL1	10.5	Naja atra	Q9DEQ3	86	14	4	99.0%	227	1.3
3FTx	Long neurotoxin 3	8.4	Naja naja	P25671	71	8	3	99.0%	108	2.1
3FTx	Neurotoxin 3	7.4	Naja sputatrix	Q9PSN6	62	29	4	99.0%	319	6.3
3FTx	Cobrotoxin-b	7.4	Naja kaouthia	P59275	61	14	4	99.0%	203	0.5
3FTx	Cytotoxin-like basic protein	7.5	Naja naja	P62377	62	7	3	99.0%	93	0.6
3FTx	Muscarinic toxin-like protein 2	7.7	Naja kaouthia	P82463	65	14	7	99.0%	210	2.2
3FTx	Weak toxin S4C11	8.0	Naja melanoleuca	P01400	65	6	2	96.9%	66	0.2
3FTx	Weak toxin CM-9a	8.0	Naja kaouthia	P25679	64	22	1	84.5%	277	5.3
3FTx	Weak neurotoxin 6	10.4	Naja sputatrix	O42256	86	46	4	99.0%	534	1.1
3FTx	Muscarinic toxin-like protein 1	7.8	Naja kaouthia	P82462	65	9	4	99.0%	109	0.7
CRISP	Cysteine-rich venom protein kaouthin-2	27.1	Naja kaouthia	P84808	238	40	10	99.0%	587	0.7
CRISP	Cysteine-rich venom protein kaouthin-1	27.8	Naja kaouthia	P84805	239	35	10	99.0%	501	2.2
CRISP	Cysteine-rich venom protein (Fragment)	39.1	Naja naja	P86543	33	22	3	99.0%	289	1.4
CRISP	Cysteine-rich venom protein TRI1 (Fragment)	27.5	Trimorphodon biscutatus	Q2XXP4	236	2	1	85.9%	24	＜0.1
CRISP	SCP domain-containing protein	15.8	Micrurus corallinus	A0A2D4G403	138	10	2	99.0%	191	0.3
NGF-beta	Venom nerve growth factor	13.4	Naja atra	P61898	116	34	10	99.0%	490	2.4
SVMP	Zinc metalloproteinase-disintegrin-like atragin	71.4	Naja atra	D3TTC2	613	63	20	99.0%	980	0.6
SVMP	Zinc metalloproteinase-disintegrin-like kaouthiagin-like	68.2	Naja atra	D3TTC1	593	44	17	99.0%	629	0.6
SVMP	Zinc metalloproteinase-disintegrin-like atrase-A	70.4	Naja atra	D5LMJ3	607	41	15	99.0%	526	0.2
SVMP	Zinc metalloproteinase-disintegrin-like mikarin (Fragments)	19.5	Micropechis ikaheca	P0DJ43	169	1	1	90.5%	15	＜0.1
SVMP	SVMP-Den-9	70.5	Denisonia devisi	R4FIC4	613	7	1	83.9%	93	＜0.1
SVMP	Metalloproteinase 12 (Fragment)	73.9	Ahaetulla prasina	A0A346CM42	614	2	2	98.5%	28	＜0.1
UP	Uncharacterized protein (Fragment)	33.5	Micrurus corallinus	A0A2D4GFB1	291	9	1	81.8%	95	1.0
UP	Uncharacterized protein (Fragment)	14.8	Micrurus surinamensis	A0A2D4Q7C6	131	4	3	99.0%	59	0.1
UP	Uncharacterized protein (Fragment)	18.8	Ophiophagus hannah	V8N885	157	3	3	99.0%	31	0.1
UP	Uncharacterized protein	40.5	Micrurus corallinus	A0A2D4FIT3	349	1	1	91.1%	15	＜0.1
GLU	Glutathione peroxidase (Fragment)	29.8	Ophiophagus hannah	V8P395	264	20	8	99.0%	277	0.6
peptidase S1	Plasminogen activator	61.6	Crotalus adamanteus	A0A0F7Z9B1	526	11	6	99.0%	148	0.1
peptidase S1	Snake venom serine protease NaSP	31.9	Naja atra	A8QL53	282	4	3	99.0%	58	0.1
peptidase S1	Tissue-type plasminogen activator (Fragment)	32.5	Ophiophagus hannah	V8NYC7	283	3	2	99.0%	45	0.1
OV	Ohanin	23.7	Phalotris mertensi	A0A182C6D0	213	7	4	99.0%	89	0.2
OV	Ohanin	24.6	Micrurus surinamensis	A0A2D4NZ30	220	4	2	95.4%	40	0.1
PDE	Snake venom phosphodiesterase	96.4	Naja atra	A0A2D0TC04	830	50	29	99.0%	730	0.2
5'-NU	Ecto-5"-nucleotidase	63.7	Micrurus tener	A0A194AS98	574	32	16	99.0%	466	0.2
FMO	Amine oxidase (Fragment)	58.3	Naja atra	A0A2R4N4Q6	507	25	19	99.0%	352	0.1
ENDOD1	Endonuclease domain-containing 1 protein	19.8	Ophiophagus hannah	V8N4Y2	160	8	6	99.0%	114	0.2
MCM	DNA helicase	97.9	Micrurus spixii	A0A2D4MKN7	863	4	1	79.3%	40	0.1
PI3/PI4-kinase	Phosphatidylinositol-4,5-bisphosphate 3-kinase catalytic subunit delta isoform (Fragment)	27.3	Ophiophagus hannah	V8NEE1	235	22	1	83.8%	230	＜0.1
S-100	Protein S100	10.3	Boiga irregularis	A0A0B8RWC4	90	1	1	89.8%	15	＜0.1
S-100	Protein S100	10.2	Micrurus fulvius	U3FBA4	90	1	1	85.8%	13	＜0.1
Type-B carboxylesterase/lipase	Carboxylic ester hydrolase	69.0	Ahaetulla prasina	A0A346CLZ4	605	4	4	99.0%	53	＜0.1
TMEM131	Transmembrane protein 131	182.2	Crotalus adamanteus	A0A0F7Z222	1592	2	2	95.3%	20	＜0.1
PLBL	Phospholipase-B 81	64.4	Drysdalia coronoides	F8J2D3	553	2	2	99.0%	40	＜0.1
Cystatin	Cystatin	16.1	Naja kaouthia	E3P6P4	141	2	1	92.7%	32	＜0.1
CSA	Cobra serum albumin	71.7	Naja naja	Q91134	614	1	1	90.6%	17	＜0.1

### Lethality and hemolytic activity of N. atra venom

**Figure 2.**
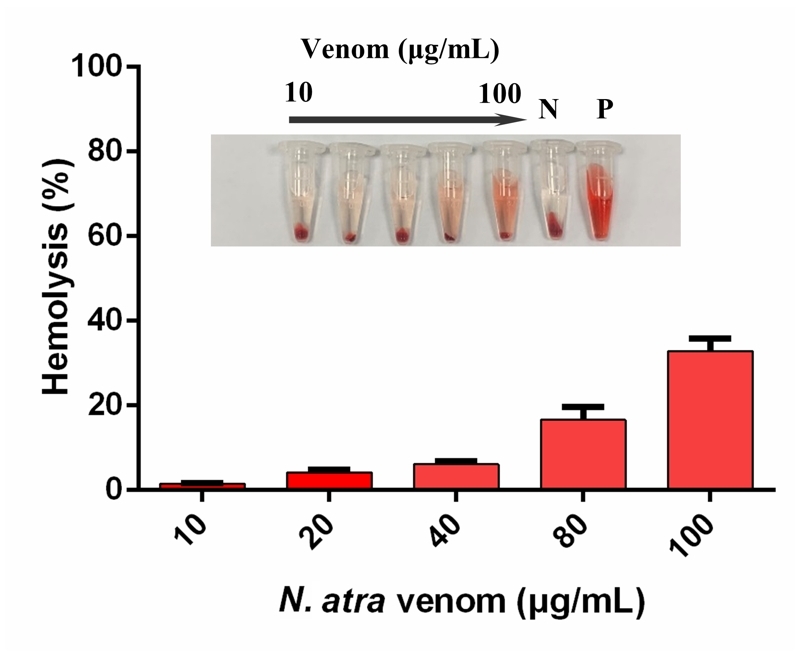
Direct hemolysis caused by N. atra venom in rabbit erythrocytes. N: negative control (saline); P: positive control (distilled water). Data are presented as mean ± SEM (n = 6 each).

Table 2 and Figure 2 shows the data on lethality and hemolytic activity of N. atra venom, respectively. The LD_50_ for Kunming mice injected subcutaneously with venom was 1.02 mg/kg, the 95% confidence interval was 0.82-1.22 mg/kg, and the clinical symptoms were muscle weakness and respiratory paralysis. As shown in Figure 2, a venom concentration of 100 ug/mL caused 32% hemolysis in rabbit erythrocytes.

**Table 2.** The median lethal dose (LD _50_) of N. atra venom.

Venom dose (mg/kg, s.c.)	Dose logarithm	Number of mice	Number of dead mice	Mortality (%)
Control	-	10	0	0
0.5	-0.3010	10	0	0
0.7	-0.1549	10	2	20
1.1	0.0414	10	6	60
1.7	0.2304	10	9	90
2.5	0.3979	10	10	100

### Histological analysis of tissue damage

**Figure 3.**
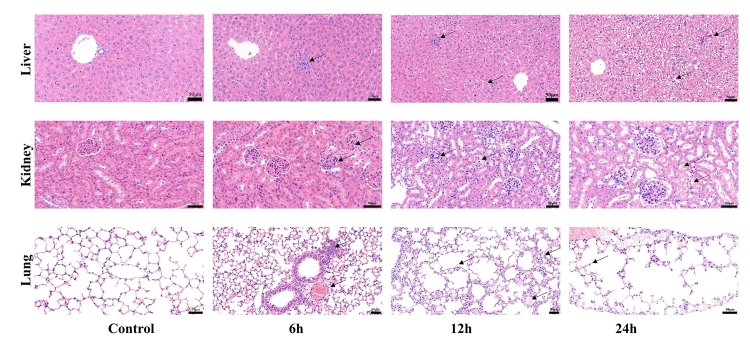
Histological analysis of hepatic, renal and pulmonary tissues of mice 6h, 12h and 24h after the injection of one LD_50_ (1.02 mg/kg, s.c.) of N. atra venom. The black arrows indicate the lesion area (scale bar: 50 μm).

Tissue sections of vital organs were stained with hematoxylin-eosin for histological analysis of venom-induced tissue damage (Figure 3). Hepatic tissues in the control groups exhibited normal cellular structures with distinct hepatic cells, hepatic portal canals, and bile ducts. However, in the experimental groups, inflammatory infiltrate (at 6h), cell border obscurity, and hepatocyte necrosis (at 12h and 24h) were clearly distinguishable. The renal tissues in the control group showed no histological abnormalities, whereas those in the experimental groups showed histological alterations such as the formation of glomerular and renal cysts (at 6h), proteinaceous material deposition in renal tubules (at 12h), epithelial cells of renal tubule edema, and cytoplasmic vacuolation (at 24h). The pulmonary tissues of control mice showed the presence of homogeneous air sacs and alveolar septa, as observed in normal lungs. However, histological alterations were noted in the lung tissues of experimental mice, including hyperplasia of peripheral lymphoid tissues and perivascular edema (at 6h), alveolar fusion, thickening and rupture of alveolar walls, and pulmonary interstitial edema (at 12h and 24h). Heart tissues of envenomed mice showed muscle fiber degeneration (see Additional file 2).

### Serum biochemical parameters

**Figure 4.**
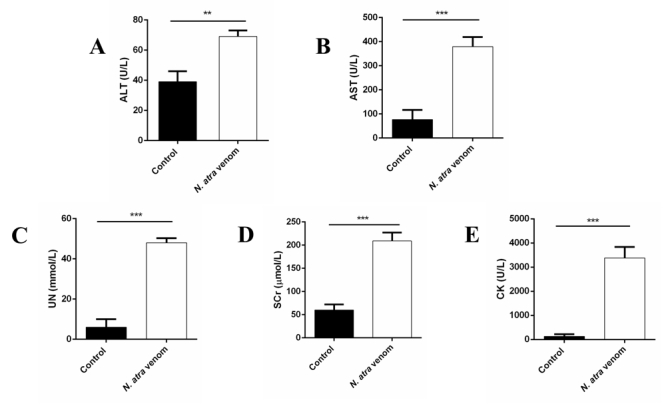
Increases in the serum **(A)** ALT, **(B)** AST, **(C)** UN, **(D)** SCr and **(E)** CK levels of mice 1h after the injection of one LD_50_ (1.02 mg/kg, s.c.) of N. atra venom. The columns represent the mean +/- SEM (n = 6 each). **p < 0.01 and **p < 0.001.

Significant increases were noted in the serum biochemical parameters of venom-injected mice compared to saline-treated (control) mice. The parameters indicative of hepatic function are shown in Figure 4A-4B: AST and ALT activities were markedly higher in the experimental group than those observed in the control group. The levels of serum UN and SCr, which are important indices of renal function, have been presented in Figure 4C-4D. CK activity, a biomarker for systemic myotoxicity, was significantly higher in envenomed miced than in the control group (Figure 4E). These parameters were markedly higher in the experimental group than those observed in the control group.

## Discussion

With growing demand for snake venom products, the economic and medicinal value of snake venom is becoming more and more prominent. However, the hidden danger is that intraspecific variation in venom composition may affect the clinical pathophysiology of envenomation, venom toxicity, and the function of venom products [23]. Hence, understanding the compositional and toxicological properties of snake venom from completely different regions is crucial. Importantly, venom used in this study was collected from the same group of snakes living in the same conditions, which is vital to guarantee a quality sample for this research [24].

In this study, SDS-PAGE of N. atra venom samples showed the presence of prominent protein bands, similar to those previously reported [4,7,8,10] and also similar to those documented for other elapids, including Naja and Bungarus caeruleus [25]. The venom proteomic results obtained here based on direct tryptic digestion supported this hypothesis. Similar to the proteomic profile of N. atra venom from other regions, such as Taiwan and Zhejiang province, China, venom proteins consisted mainly of 3FTx and PLA_2_ [2,8,26,27]. However, the relative abundances of these two families of toxins were not significantly different from each other, in contrast to previous studies [2,8,26]. Geographic variation in N. atra venom proteomic profiles has been observed, with 3FTx showing a surprising geographical variation [8]. However, these studies have focused mainly on factors that influence venom variation, an aspect that we did not address in our study. Future studies should examine the factors that influence intraspecific variation in venom composition and the mechanisms involved. 

Consistent with previous work, the main component of 3FTx in N. atra venom is cytotoxin, known as cardiotoxin, followed by short chain α-neurotoxin, weak neurotoxin and long chain neurotoxin [2,8,28]. Cytotoxins are the principal toxic components that cause hemolytic damage, membrane disruption, and circulatory failure [29]. Cardiotoxin isolated from N. atra venom induces direct hemolysis in washed erythrocytes from several animals [30]. Although we were unable to identify a single specific component of venom for inducing hemolysis, we did find that N. atra venom induce direct hemolysis in rabbit erythrocytes in the present study, unlike other cobra venoms [31].

Furthermore, the short-chain α-neurotoxin (type I α-neurotoxin) toxin of 3FTx in this venom binds to the muscle nAChR and inhibits the binding of acetylcholine to its receptor, thereby impairing neuromuscular transmission [32]. Orphan group II toxins showed weak α-neurotoxin activity. Long-chain α-neurotoxin (type II α-neurotoxin) of 3FTx is a peripheral nAChR antagonist that causes paralysis, respiratory failure, and death [33,34]. Overall, based on venom proteomics, N. atra venom would exhibit strong neurotoxicity in experimental animals [2]. In agreement with this, the lethality assay showed that the s.c. injection of N. atra venom caused muscle weakness and respiratory paralysis. However, for humans, neurotoxic symptoms do not seem to be a clinically vital consequence of N. atra envenomation [3]. The reason for the discrepancy between neurotoxic effects of N. atra envenomation in mice and humans remain unclear. Moreover, the LD_50_ of venom in the present study was 1.02 mg/kg, which was higher than that of venom samples collected from Zhejiang (0.68 mg/kg, s.c.) and Taiwan (0.29 mg/kg, s.c.) [2,8]. Although the origin of the venom is different, the lethal dose of venom in this study was higher than that of other regions, suggesting that it might be less lethal. 

PLA_2_ is the most abundant component of venom in this study, indicating that it may be an important factor in the pathogenesis of N. atra envenomation. PLA_2_ exert multiple toxic effects on the prey or victim, such as membrane damage, myotoxicity, neurotoxicity, and edema [35,36]. Moreover, PLA_2_ has also been implicated in multiple pathologies, including hepatotoxicity and nephrotoxicity [37]. Additionally, PLA_2_ from Naja kaouthia venom has been reported to promote CTX-induced cytotoxicity in cell models, leading to cell vacuolation and rupture [27], which indicates that cobra PLA_2_ play a role in the pathogenesis of necrosis. N. atra venom triggered considerable cell necrosis in the hepatic, renal and pulmonary tissues of mice in this study, which was similar to the findings reported for venom collected from other cobra species [38,39]. Importantly, the significant changes in serum biochemical parameters observed here indicated that N. atra venom caused damage to the liver, kidney, and lung of the mice.

Myonecrosis is a common phenomenon in animals injected with snake venom [40]. As shown in the present study, a significant increase in serum levels of CK also indicated that skeletal and cardiac muscle necrosis occurred in the mice injected with N. atra venom, and similar activity was reported in other studies conducted on different types of snake venom [41-43]. Several previous studies have reported the occurrence of muscle fiber degeneration after the injection of cardiotoxin from N. atra [44-46], which is similar to the pathological analysis results of heart tissue obtained in the present study. The study of Naja venom is important due to its great potential for medical application as an anticancer among others [47].

## Conclusion

In conclusion, the present study investigated the components of farm-raised N. atra venom and assessed its toxicity in vivo and in vitro. This work extends our knowledge regarding venom of farm-raised N. atra, indicating that PLA_2_ and 3FTx are major protein families in venom that exhibit direct hemolytic activity, neurotoxicity, myotoxicity, and induce pathological changes in tissues. These findings provide information on the venom of captive N. atra from Hunan province, China, that should be useful in future comparisons with venom from wild snakes from the same geographic region.

### Abbreviations

3FTx: three-finger toxin; 5ʹNUC: 5ʹ-nucleotidases; ALT: alanine aminotransferase; AST: aspartate aminotransferase; CID: collision induced dissociation; CK: creatine kinase; CRISP: cysteine-rich secretory protein; CSA: cobra serum albumin; CVF: cobra venom factor; ddH_2_O: double distilled H_2_O; DTT: dithiothreitol; HPLC, high-pressure liquid chromatography; ENDOD1: endonuclease domain-containing 1 protein; ESI: electrospray ionization; FMO: flavin monoamine oxidase; GLU: glutathione peroxidase; H&E: hematoxylin and eosin; KUN: Kunitz-type serine protease inhibitors; LAAO: L-amino acid oxidase; LD_50_: median lethal dose; LNTX: long-chain α-neurotoxin; N. atra: Naja atra; Nano-LC-ESI-MS/MS: nano liquid chromatography with electrospray ionization tandem mass spectrometry; OV: ohanin/vespryn; PDE: phosphodiesterase; PLA_2_: phospholipase A_2_; PLBL: phospholipase B-like; SCr: serum creatine; SDS-PAGE: sodium dodecyl sulfate-polyacrylamide gel electrophoresis; SNTX: short-chain α-neurotoxin; SVMP: snake venom metalloproteinase; UN: urea nitrogen; UP: uncharacterized protein; WNTX: weak neurotoxin.

## Supplementary material

The following online material is available for this article:

Additional file 1. Identification results of nano-LC-ESI-MS/MS protein spectrum of *N. atra* venom.

Additional file 2. Histological analysis of heart tissue from mice 24h after the injection of one LD50 (1.02 mg/kg, s.c.) of N. atra venom. The black arrows indicate the lesion area (scale bar: 50 μm).
