# Supplementary material for: Compositional and toxicological investigation of pooled venom from farm-raised Naja atra
Source: J Venom Anim Toxins Incl Trop Dis. 2022 Mar 14;28:e20210040. doi: 10.1590/1678-9199-JVATITD-2021-0040 (PMC8956250; doi:10.1590/1678-9199-JVATITD-2021-0040)
Supplement: Additional file 2. [file 1678-9199-jvatitd-28-e20210040-s2.pdf]

J Venom Anim Toxins incl Trop Dis
https://doi.org/10.1590/1678-9199-JVATITD-2021-0040

Supplementary Material to "Compositional and toxicological investigation of pooled venom from farm-raised *Naja atra*"

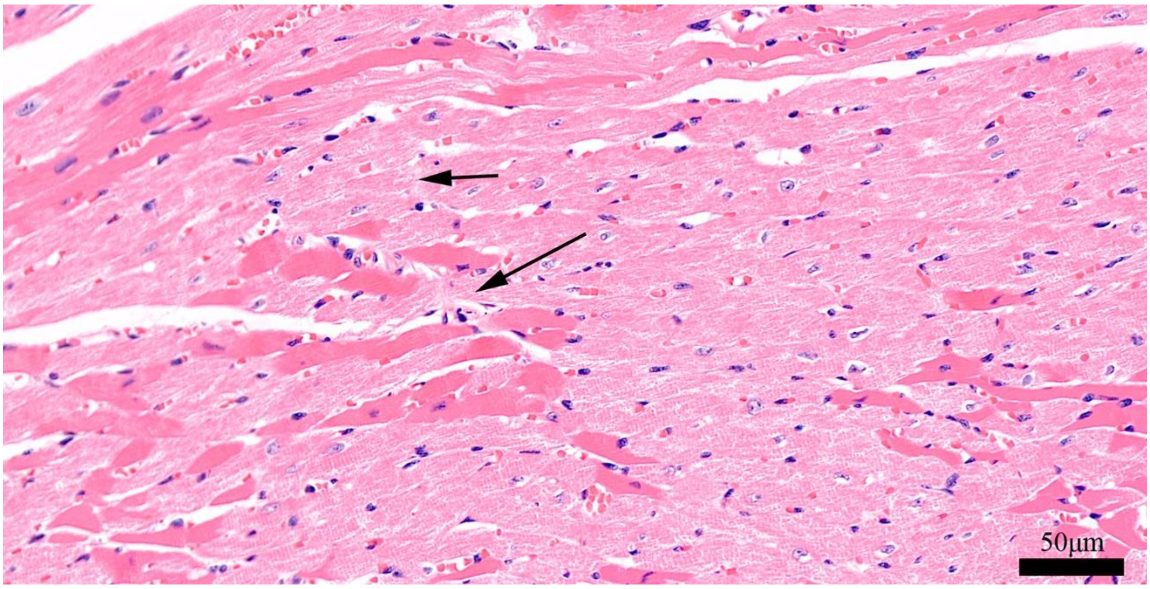

Additional file 2. Histological analysis of heart tissue from mice 24h after the injection of one LD_{50} (1.02 mg/kg, s.c.) of *N. atra* venom. The black arrows indicate the lesion area (scale bar: 50 μm).
